# Cross-modal and modality-specific expectancy effects between pain and disgust

**DOI:** 10.1038/srep17487

**Published:** 2015-12-03

**Authors:** Gil Sharvit, Patrik Vuilleumier, Sylvain Delplanque, Corrado Corradi-Dell’Acqua

**Affiliations:** 1 Swiss Centre for Affective Sciences, University of Geneva, CH-1211 Geneva, Switzerland; 2 Laboratory for Neurology and Imaging of Cognition, Department of Neurosciences and Clinic of Neurology, University Medical Centre, CH-1211 Geneva, Switzerland; 3 Department of Psychology, FPSE, Unviersity of Geneva, CH-1211 Geneva, Switzerland

## Abstract

Pain sensitivity increases when a noxious stimulus is preceded by cues predicting higher intensity. However, it is unclear whether the modulation of nociception by expectancy is sensory-specific (“modality based”) or reflects the aversive-affective consequence of the upcoming event (“unpleasantness”), potentially common with other negative events. Here we compared expectancy effects for pain and disgust by using different, but equally unpleasant, nociceptive (thermal) and olfactory stimulations. Indeed both pain and disgust are aversive, associated with threat to the organism, and processed in partly overlapping brain networks. Participants saw cues predicting the unpleasantness (high/low) and the modality (pain/disgust) of upcoming thermal or olfactory stimulations, and rated the associated unpleasantness after stimuli delivery. Results showed that identical thermal stimuli were perceived as more unpleasant when preceded by cues threatening about high (as opposed to low) pain. A similar expectancy effect was found for olfactory disgust. Critically, cross-modal expectancy effects were observed on inconsistent trials when thermal stimuli were preceded by high-disgust cues or olfactory stimuli preceded by high-pain cues. However, these effects were stronger in consistent than inconsistent conditions. Taken together, our results suggest that expectation of an unpleasant event elicits representations of both its modality-specific properties and its aversive consequences.

When people make predictions, they may experience subsequent events differently than novel and unexpected happenings. Neurophysiological studies show that expectations improve perceptual sensitivity by selectively activating those neuronal populations involved in processing specific features such as colour, orientation, etc.^1^, a phenomenon at the root of endogenous attention. Accordingly, expectations of auditory, tactile, visual, or olfactory stimuli increase the activity in corresponding sensory-specific cortices^2^,^3^. Expectancy effects are also observed in the domain of pain^4^: cues predicting high intensity stimulations increase the subjective pain experience (as tested through explicit ratings)^5^,^6^,^7^,^8^,^9^.

However, pain is a multi-component experience that has both sensory and affective aspects, with intrinsic and strong unpleasantness. It could be argued that pain expectancy elicits modality-specific information about the predicted event, as for the case of vision, audition, etc. For instance, tactile stimuli are perceived more rapidly when occurring at the same location (right *vs*. left hand) at which pain is expected^10^, thus implicating information about the affected body segment. Furthermore, neuroimaging studies suggest that expectations of pain re-enact the same neural network (somatosensory cortex, insula and cingulate cortex, etc., also known as the *pain matrix*^11^,^12^) involved in the real experience of pain^5^,^6^,^7^.

Alternatively, it could be hypothesized that pain expectancy elicits information about the negative affective consequence of predicted events, implying an amodal or supramodal representation shared with other unpleasant, non-painful stimuli. For instance, pain shares many important features with the experience of disgust, as they are both unpleasant, arousing, relevant for one’s survival, intimately linked to interoceptive processing^13^, and eliciting similar facial expressions^14^. In addition, it has been reported that the perceived unpleasantness of a painful event can be modulated by the valence of a concurrent olfactory stimulus^15^,^16^, suggesting that the two modalities might engage a shared affective dimension. Furthermore, brain areas associated with the *pain matrix* might actually not code the sensory-specific components of pain experience, but process a wider range of sensory and affective events^17^,^18^,^19^, which might be relevant or salient for individuals’ goals^20^,^21^,^22^, including disgust^23^,^24^,^25^,^26^,^27^. It therefore remains unclear whether expectation of pain holds information related to the sensory component of the stimulus or its affective consequences (potentially common with other unpleasant stimuli such as disgust).

To address this crucial issue, we investigated how expectations of either pain or disgust influence the experience of different, but comparably unpleasant, stimulations: thermal (painful) and olfactory (disgusting). We presented to participants cues predicting both the unpleasantness (high/low) and the modality (pain/disgust) of upcoming events, and asked them to rate the subjective unpleasantness of a subsequent thermal or olfactory stimulus (Fig. 1A). Critically, unpleasantness of stimuli was carefully matched between modalities to ensure that, despite the many qualitative differences between pain and disgust, these could be comparable at least under this dimension. Based on earlier findings^5^,^6^,^7^,^8^,^9^, we hypothesized that cues threatening high (relatively to low) pain would increase the subjective ratings of subsequent thermal stimulations (expectancy effect); likewise, cues predictive of high-disgust should increase the subjective ratings of subsequent olfactory stimulations. The critical condition, however, concerned stimuli preceded by cues in the inconsistent modality (odours preceded by high-pain cues, and temperatures preceded by high-disgust cues), which allowed us to test for cross-modal expectancy effects. If cues trigger a modality-specific representation of the upcoming stimulus, then the perceived unpleasantness should not be affected by inconsistent cues; however, if cues trigger a supramodal representation of unpleasantness/aversiveness, then both the consistent and inconsistent cues should elicit reliable expectancy effects. In addition to this main hypothesis, as expectancy effects (either modality-specific or modality independent) are reflective of the degree to which individuals trust the experimental set-up, we assessed also the effect played by personal reliance on the information provided by the cue, as measured by a post-experimental questionnaire. In addition, although our main interest was to investigate explicit subjective ratings (as previous studies on pain expectancy^5^,^6^,^7^,^8^,^9^), we also recorded autonomic measures (heart rate, electrodermal activity, respiration) to obtain more objective measures of emotional arousal^6^,^8^,^9^.

## Materials and Methods

### Participants

We recruited 18 participants (10 females, mean age 26.28 years). None had any history of neurological/psychiatric illness, reported any olfactory deficit. Written informed consent was obtained from all subjects, who were naive to the purpose of the study. The study was approved by the local ethics committee (*Commission centrale d’éthique de la recherche sur l’être humain des HUG* [Central commission of ethics in research on human beings of the University Hospital of Geneva]) and conducted according to the declaration Helsinki.

### Olfactory stimulation

Odorants were delivered to the subjects’ nostrils by means of rubber cannulas connected to a computer-controlled, multi-channel, custom-built olfactometer. The olfactometer is able to reliably release various kinds of compounds over multiple trials, without contamination from one trial to the other, at known times, and without additional noise or tactile stimulation in the nose (technical details for this apparatus have been described elsewhere^28^). Odorous substances were diluted at variable concentrations in odourless dipropylene glycol. Odours were embedded in 1 l/min constant and filtered airstream. Four odorants provided by Firmenich, SA (Geneva) were selected on the basis of previous evaluations^29^,^30^. *Isovaleric acid* (evoking dirty socks) and *gee* (evoking rotten food) were chosen to reliably elicit disgust in the participants. Each of these two substances was diluted in five different concentrations (0.1%, 0.5%, 1%, 5% and 10%). In addition, lilac (10%) and soap (10%) odours were also used to elicit positively valenced sensations in participants. These positive odours were used to give to participants an occasional relief from the disgusting odours and to reduce the occurrence of habituation or sensitization effects. Thus, our experimental set-up included 12 olfactory stimuli (*isovaleric acid* and *gee*, each at 5 different concentrations, plus *lilac* and *soap*), as well as a 13^th^ odourless solution of dipropylene glycol that served as a control.

In the main experiment, each participant was presented with 4 out of the possible 12 olfactory stimulations. These comprised one odorant expected to elicit disgust, at three different concentrations: low (unpleasantness ratings ~ −5), medium (~ −20), and high (~ −40). Additionally, a fourth odorant was expected to elicit a pleasant experience. Those four odours were selected, at the individual level, on the basis of a pleasantness-rating task carried out at the beginning of the experimental session (see Supplementary Materials for more details).

### Thermal stimulation

Thermal stimuli were delivered through a computer-controlled thermal stimulator with a 25 × 50 mm fluid-cooled Peltier probe (MSA Thermal Stimulator–Somedic AB, Sweden). The stimulator was attached to one of the participant’s leg. Given that the main experimental session was divided into four blocks, each block alternated between simulation of one and the other leg, thus minimizing habituation/sensitization to the thermal stimuli. Thus, for each participant and each leg, we selected three different temperatures that aimed at evoking three different levels of pain: low (on average 44.39 °C), medium (45.62 °C) and high (46.85 °C). Critically, we made sure that the unpleasantness associated with these three temperatures was comparable to that of the three disgusting odours selected for the same participant. See Supplementary Materials for more information on the temperature selection method.

## Main Experimental Setup

### Task Design

**Figure 1:**
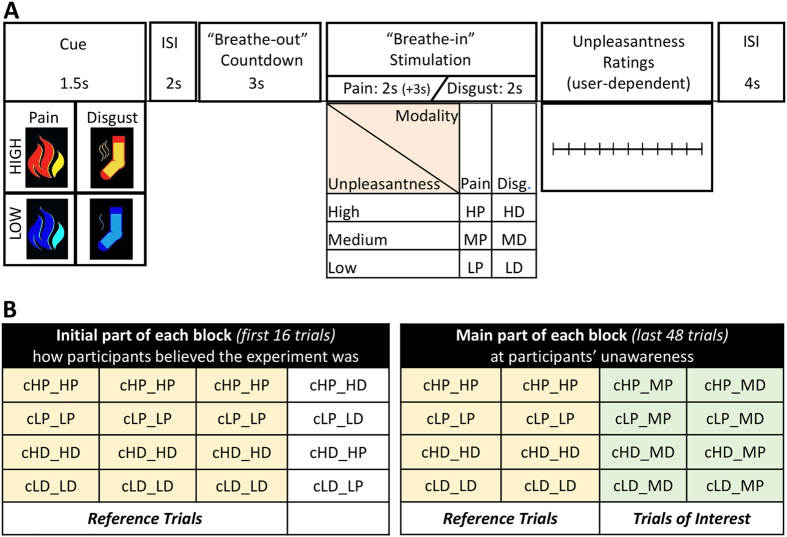
(**A**) Trial Structure. Each trial began with one pictorial cue presented for 1.5 s and followed by an inter-stimulus interval (ISI) of 2 s. Participants were then instructed to “breathe-out” during a 3 s countdown. Then, a “breathe-in” instruction appeared together with stimulus delivery – which could be either olfactory or thermal. All stimuli lasted 2 s (additional 3 s were necessary for thermal stimuli to reach the target temperature). Stimuli were followed by a visual analogue scale for self-paced unpleasantness ratings. Four different kinds of cues were presented, predicting the unpleasantness (high/low) and modality (pain/disgust) of the upcoming stimulation (thermal pain/olfactory disgust). (**B**) Structure of each of the four experimental blocks. Participants were told that cues were 100% of the times predictive of the unpleasantness of the upcoming stimulus, and 75% of its modality (i.e., in 1 trial out of 4 pain cues were followed by olfactory stimuli, or disgust cues were followed by thermal stimuli). The trials relevant for testing expectancy effects (“Trials of interest”) were those in which, at participants’ unawareness, thermal and olfactory stimulations were aimed at eliciting moderate pain/disgust, regardless of the information provided by the preceding cue. To maintain participants’ belief in the cue-stimulus association, trials of interest were intermingled with “Reference Trials”, in which the stimulation was correctly predicted by the cue. Full details in the Methods section.

The main experimental task was organized in four blocks. On each trial, a 1.5 s predictive cue was presented and followed by a 2 seconds (s) anticipatory interval with a fixation cross at the screen centre (see Fig. 1A). Next, a 3 s countdown period was presented with the instruction “Breathe-out” written on the screen. Once the countdown reached 0, a 1 s “Breathe-in” instruction appeared together with either a thermal or olfactory stimulation. Both olfactory and thermal stimuli lasted 2 s, although additional 3 s were necessary for the thermal stimulator to reach the target temperature. Participants were prompted to breathe according to the instructions. After the stimulation, participants rated its unpleasantness on a visual analog scale using the hand-held mouse. The scale remained on the screen until a response was delivered and followed by a 4 s inter-stimulus interval. On the basis of the olfactory and thermal stimuli selection sessions, we selected two comparable medium-unpleasant stimuli for each modality: *painful* (*MP*) and *disgusting* (*MD*). Each of these two stimuli were presented following four possible types of cues, which were expected to trigger expectations of *high-pain (cHP)*, *low-pain (cLP)*, *high-disgust (cHD),* and *low-disgust* (*cLD*). The cues were schematic representations of either a smelly sock (predicting disgusting odours) or a flame (predicting painful temperatures) (see Fig. 1A). This yielded eight balanced conditions of interest. Half were modality-consistent (pain cue followed by medium pain: *cHP_MP* or *cLP_MP*; disgust cue followed by medium disgust: *cHD_MD* or *cLD_MD*). The remaining half were modality-inconsistent (i.e., *cHP_MD*, *cLP_MD*, *cHD_MP* and *cLD_MP*). Importantly, participants were not informed that the thermal/olfactory stimulations occurred with a fixed medium-unpleasantness, but were instructed that the stimulations would correspond to the unpleasantness (“in all of the cases”) and the modality (“in 3 out of 4 of the cases”) described by the cues. This instruction was made plausible by the addition of trials of no interest (“Reference trials”), in which low- or highly- unpleasant stimulations were delivered instead of the medium ones (Fig. 1B).

The structure of each block is fully described in Fig. 1B. In the first 16 trials, participants received (in random order) 12 “reference” trials that were consistent with the preceding cue both in terms of unpleasantness and modality (3 trials *cHP_HP*, 3 trials *cLP_LP*, 3 trials *cHD_HD* and 3 trials *cLD_LD*) and 4 trials that were consistent with the preceding cue only in terms of unpleasantness but not in terms of modality (1 trial *cHP_HD*, 1 trial *cLP_LD*, 1 trial *cHD_HP* and 1 trial *cLD_LP*). Thus, in 75% of the trials a cue informing about a given modality was followed by a stimulation of the same modality, whereas in the remaining 25% of trials the stimulation was in a modality opposite to the prediction of the cue. However, the cue was always predictive of the unpleasantness of the subsequent stimulus. In the last 48 trials, participants received 24 “reference” trials consistent with the preceding cue in both unpleasantness and modality (6 trials *cHP_HP*, 6 trials *cLP_LP*, 6 trials *cHD_HD* and 6 trials *cLD_LD*), and 24 trials in which high/low stimulations were replaced by medium unpleasantness stimuli according to the 8 conditions described above (3 trials *cHP_MP*, 3 trials *cLP_MP*, 3 trials *cHD_MP*, 3 trials *cLD_MP,* 3 trials *cHP_MD*, 3 trials *cLP_MD*, 3 trials *cHD_MD* and 3 trials *cLD_MD*). These 24 medium trials were the main objective of the experiment. Keep in mind that, if the unpleasantness of medium trials were to be modified to match that announced by the preceding cue, participants would have faced the same cue predictability pattern (100% predictive of the unpleasantness, 75% of the modality) as in the first part of the block. This design structure suits well our purposes as it provides cues that are highly predictable in all their dimensions (unpleasantness, modality), and yet allows studying the effects of these predictions on a balanced subset of medium-unpleasant stimuli.

Finally, in each block, these 64 trials (16 + 48) were further intermingled with 13 trials in which the positive odour was administered following a corresponding cue (schematic flower). Each experimental block lasted about 21 minutes. Stimulus presentation was controlled using Cogent 2000 (Wellcome Dept., London, UK), as implemented in Matlab R2012a (Mathworks, Natick, MA).

### Procedure

Participants seated in a lab-chair in front of a computer screen. First, they were requested to report to what extent their subjective experience to each pictorial cue (see Fig. 1) matched 9 emotions (anger, fear, relief, anxiety, happiness, stress, boredom, irritation, and disgust) on a scale from 1 (not at all) to 5 (absolutely). The same rating task was then repeated at the end of the experimental session. The comparison of pre vs. post-experiment ratings was used to provide an indirect manipulation check, allowing us to assess which kinds of emotion were significantly modulated by the task, regardless of any pre-existing effect elicited by the cue stimuli. Next, a battery of questionnaires (STAI^31^, SPQ^32^, PANAS-X^33^ and CES-D^34^) was acquired to capture trait-related individual variability that might impact the performance in the expectancy task.

Participants were then connected to both the olfactometer and the thermode, and underwent the olfactory and thermal stimuli-selection sessions (as described in supplementary materials), followed by the main experimental session (in four blocks). After completion of the experiment, participants were debriefed through ad hoc questionnaires aimed at measuring task-related individual variability, such as the degree to which they relied on the cues. Such debrief allowed us to assess whether individuals who might distrust our experimental set-up would exhibit weaker expectancy effects.

### Physiological recordings

Electrodermal activity, heart rate, and respiration were recorded throughout the experiment using the MP150 Biopac Systems (Santa Barbara, CA) with a 1000 Hz sampling rate. Electrodermal activity was measured with Beckman Ag–AgCl electrodes (8-mm diameter active area) filled with a skin conductance paste (Biopac) attached to participants’ non-dominant hand on the palmar side of the middle phalanges of the second and third fingers. Skin conductance responses (SCRs) were measured in microSiemens and analyzed offline (Lowpass filter: 5 Hz). We measured the artefact-free amplitude of SCRs evoked by stimulations. Following previous recommendations^35^,^36^, we considered a reliable SCR amplitude as exceeding a threshold of 0.02 μS that started between 1 and 4 s after stimulation, and peaked in the period between this onset time and 8 s after stimulation. The resulting amplitude value was log-transformed.

Heart rate (HR) was assessed by fixing the Biopac pregelled disposable electrodes under the participants’ left and right clavicles and on the left waist. The signal was band-pass filtered (between 10–30 Hz) and electrocardiographic R waves were detected offline; then intervals between heartbeats were converted into HR, expressed in beats per minute. HR values within each trial were normalized with a baseline average HR value recorded in the 2.5 s preceding the cue presentation. In the analysis of HR, we took into account the strong relationship between cardiac responses and respiration: more specifically, it has been shown that HR variations in cued-sniffing trials (as used in our paradigm) are characterized by a first increase of HR, in temporal proximity with inspiration onset, and a subsequent cardiac deceleration^36^. Critically, the HR values following the deceleration phase were found to be modulated by the hedonicity of the sniffed odorant, with higher HRs for more unpleasant odours^36^. We therefore followed this model and focused our analysis on that aspect of the respiration-induced cardiac response which is most sensitive to unpleasantness, as indexed by the lowest HR value between 5 and 8 s after the inspiration onset^36^.

Finally, respiratory activity was recorded through a 2.5 mm tube (interior diameter) positioned at the entrance of the participants’ right nostril, on the nasal cannula used to deliver the odorants, which was connected to a differential pressure transducer (TSD160A;  ± 2.5 cm H2O sensitivity range) to continuously record variations in the nostril airflow. To investigate whether participants exhibited differential breathing patterns across different stimulus conditions, we identified in each individual trial those inspirations (sniffs) that occurred within the first two seconds from the stimulus onset (corresponding to the time in which odorants were delivered during the olfactory stimulation). We then calculated, as estimate of overall inspiration volume of each trial, the area under the curve (AUC) of all the identified sniffs.

## Results

### Behavioural Ratings

As first step, we focused on the “reference trials” (see Fig. 1B), in which the cue was consistent with all dimensions of the subsequent stimulation (i.e., *cHP_HP*, *cLP_LP*, *cHD_HD*, *cLD_LD*). For each subject, we excluded those blocks in which the “reference” trials with high pain or high disgust stimuli were rated as neutral (*cHP_HP* ≥ −5 or *cHD_HD* ≥ −5), or considered as equally (or more) pleasant than the low pain or low disgust stimuli, respectively (*cHP_HP* ≥ *cLP_LP* or *cHD_HD* ≥ *cLD_LD*). As a result, 5 out of 72 blocks (18 subjects * 4 blocks per subject) collected in the whole study were excluded from the final analysis (6.9%), thus insuring that, for the remaining part of the data, high pain/disgust stimuli were indeed experienced as more unpleasant than their corresponding control conditions. Keep in mind, however, that blocks were never discarded on the basis of data from the medium stimulation trials.

Our main goal indeed was to test for the influence of high *vs*. low cues on participants’ ratings for stimuli from either the same or different modalities, while stimulation intensity was actually kept constant (at medium intensity). To this aim, the ipsatized (*z*-transformed) ratings^37^ of subjective unpleasantness experienced on medium trials were submitted to a repeated-measure ANCOVA with Cue Unpleasantness (high vs. low), Cue Modality (pain vs. disgust), and Stimulus Modality (painful vs. disgusting) as factors. As covariate, we added a measure of conscious beliefs from the post-experimental debrief, evaluating the degree to which a given participant ignored or relied on the cue (based on the item “The cue was too unpredictable, I ignored it most of the times”, rated on a Likert scale from 1 [totally disagree] to 5 [totally agree]). This covariate was included as we wished to inspect if individuals who trusted the most our experimental set-up were more susceptible to expectancy effects.

**Figure 2:**
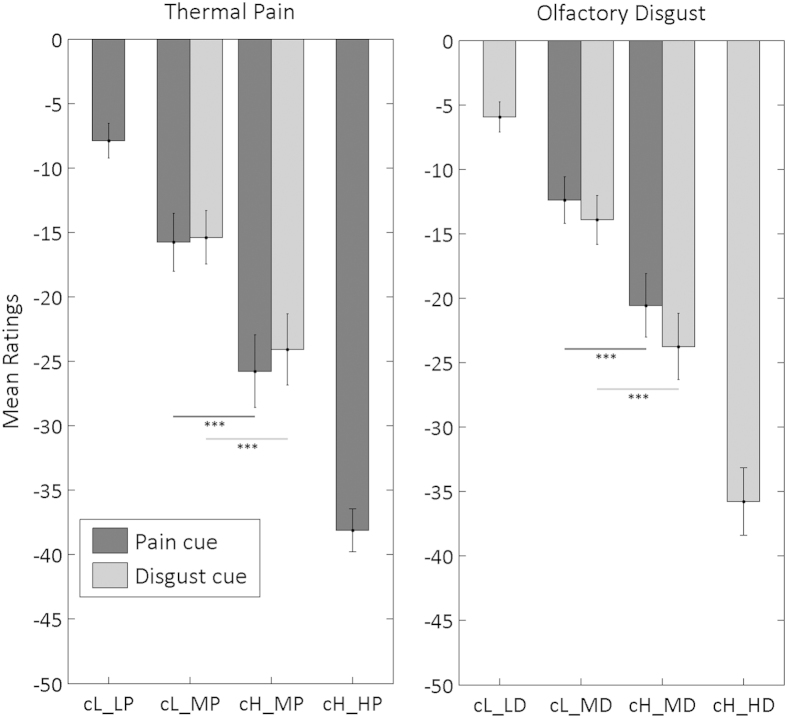
Unpleasantness ratings associated with thermal (left side) and olfactory (right side) stimuli as a function of the predictive cues. Data averaged across 18 subjects. The more values are negative, the more unpleasant was the experience. Dark grey bars refer to stimuli preceded by a pain cue, light grey bars to those preceded by a disgust cue. The different combinations of cues and stimuli are labelled as follows: cL and cH = cue for low and high pain/disgust; LP, MP, and HP = thermal pain stimuli of low, medium, and high unpleasantness; LD, MD, and HD = olfactory disgust stimuli of low, medium, and high unpleasantness. Error bars refer to standard errors of the mean. ***refer to conditions eliciting differential unpleasantness at p < 0.001 (see results). Please note that the figure displays raw rating values for readability purposes (statistical analysis was run on *z*-transformed data).

This analysis revealed no main effect of Cue Modality (F_(1,16)_ = 0.54, non significant [*n.s.*]) or Stimulus Modality (F_(1,16)_ = 2.07, *n.s.*), confirming that our stimulus sets were well matched. Instead, a highly significant main effect of the Cue Unpleasantness was found (F_(1,16)_ = 23.33, P = 0.0002), reflecting higher unpleasantness ratings for identical (medium unpleasantness) stimuli when they followed a high rather than a low unpleasantness cue (see Fig. 2 and Table 1). This global expectancy effect was further explored through post-hoc Bonferroni-corrected *t*-tests, examining each of the four possible combinations of cue and stimulus modality (critical p-value = 0.05/4 = 0.0125). In line with previous findings^5^, we found a significant increase of unpleasantness for pain when a medium thermal stimulation was preceded by a high-pain (relative to low-pain) cue (*cHP_MP* > *cLP_MP:* t_(17)_ = −4.73, P = 0.0002). Furthermore, the same increase of unpleasantness was observed when a medium disgust stimulation was preceded by a high-disgust (relative to low-disgust) cue (*cHD_MD* > *cLD_MD:* t_(17)_ = −4.06, P = 0.0008), extending expectancy effects previously observed for pain^5^ to another modality. Most critically, such expectancy effects were also found in the cross-modal conditions, that is when the modality of the cue was inconsistent with the modality of the stimulus (see Fig. 2 and Table 1; *cHD_MP* > *cLD_MP:* t_(17)_ = −4.89, P = 0.0001;*cHP_MD* > *cLP_MD:* t_(17)_ = −3.47, P = 0.003).

When testing the interaction terms in the ANCOVA, we found neither a significant interaction between Cue Modality and Cue Unpleasantness (F_(1,16)_ = 0.002, *n.s.*) nor between Stimulus Modality and Cue Unpleasantness (F_(1,16)_ = 0.02, *n.s.*), indicating that these expectancy effects had a similar magnitude in the pain and disgust domain. Instead, we found a significant interaction between Cue Modality and Stimulation Modality (F_(1,16)_ = 5.94, P = 0.027), reflecting stronger unpleasantness for equal (medium) stimuli when the Stimulus Modality was consistent with the Cue Modality. Furthermore, this differential effect of consistent cues was primarily driven by trials with a high-intensity cue (see also Fig. 2 and Table 1), as indicated by the significant three-way interaction Cue Unpleasantness * Cue Modality * Stimulus Modality (F_(1,16)_ = 4.81, P = 0.043). Please note that all the effects described above were also observed when removing the covariate from the analysis (except for the three-way interaction which led to only marginal significance: F_(1,17)_ = 3.73, P = 0.070).

**Figure 3:**
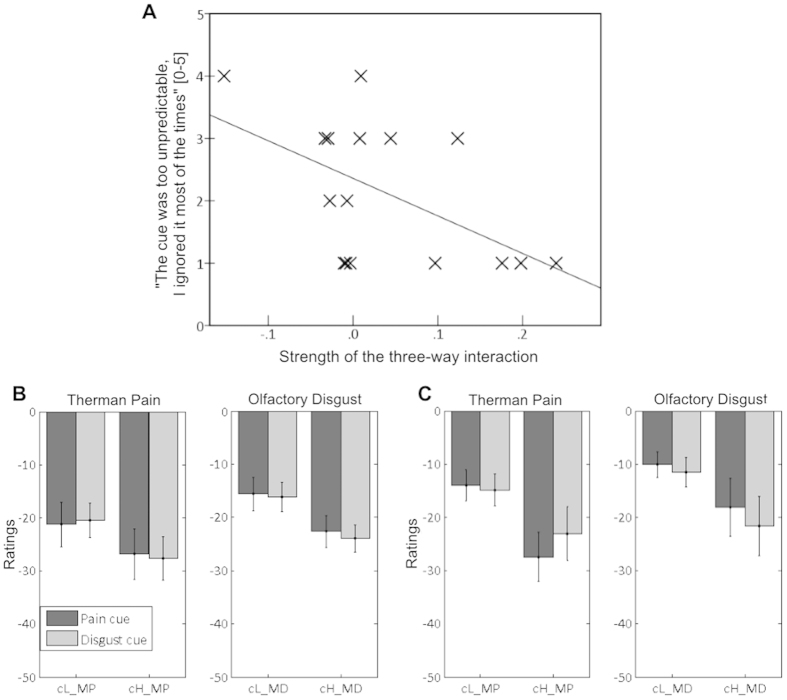
Influence of conscious reliance on the cue on the strength of modality-specific expectancy effects (three-way interaction). (**A**) Reliance on the cue, as measured in the post-experimental debrief, is plotted against the strength of the three-way interaction Cue Unpleasantness*Cue Modality*Stimulus Modality, which reflects stronger expectancy effects (cH > cL) in consistent (same modality) as opposed to inconsistent (different modality) medium trials. Higher values on the vertical axis refer to participants who ignored the cue most of the time. Higher values on the horizontal axis refer to stronger modality-specific cue effects. For visualization purposes of the covariate effect, we display (**B**) the unpleasantness ratings for half of the participants who ignored most the cues (defined through median split of the individual covariate values) and (**C**) the half who relied mostly in them. Axis and bar codes in subplots B and C are similar to those in Fig. 2.

We then tested if any of the factors in the ANCOVA interacted with the covariate measuring conscious beliefs. Only the three-way interaction Cue Unpleasantness * Cue Modality * Stimulus Modality was found to be modulated by beliefs (leading to four-way interaction Stimulus Modality * Cue Unpleasantness * Cue Modality * Belief covariate – F_(1,16)_ = 5.89, P = 0.027). Figure 3A displays the strength of this three-way interaction in each participant, plotted against the covariate: this clearly shows that the more participants ignored the cue, the less the three-way interaction was present, a pattern confirmed by visual inspection of Fig. 3B,C which illustrates the unpleasantness ratings from half of the participants who mostly ignored the cue, together with ratings from the other half who relied on it. This pattern indicates that the modality-specific increase in anticipatory effects (relative to cross-modal effects) was at least partly determined by the degree to which participants relied on the cues and believed in their predictive value.

Finally, we also tested for any influence of other individual scores, using measures from questionnaires on personality traits or other ad hoc items in the post-scanning debrief questions (e.g., “Rating the odours unpleasantness was very difficult for me”, “I often lost concentration during the four sessions”). These data were submitted to exploratory ANCOVAs similar to the one described above. In none of these analyses, the individual covariates were associated with significant effects, neither under a rigorous Bonferroni-correction for the number of ANCOVAs employed, nor when adopting a more liberal uncorrected approach.

### Physiological measures

To support our explicit rating results with additional indices for the impact of expectancy on affective responses to pain or disgust stimuli, we also recorded objective physiological parameters reflecting emotional arousal, including skin conductance response (SCR), hearth rate (HR), and respiration (inspiration air volume). Modulations of autonomic responses along any of these three measures were considered as significant when surviving a statistical threshold with a Bonferroni-corrected p-value ≤ 0.0167 (corresponding to 0.05/3).

**Figure 4:**
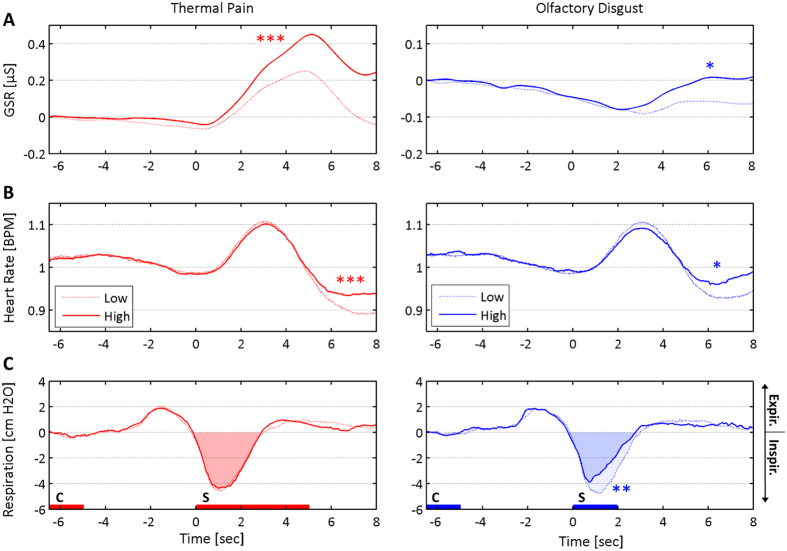
Event-related physiological responses during the reference trials (cue fully consistent with stimulation). (**A**) Skin conductance response (**B**) heart rate, and (**C**) respiration averaged across 18 subjects. Data for thermal stimulation in the left column, olfactory stimulation in the right column. The horizontal axis reflects time in each trial, with 0 referring to the stimulation onset (marked as *S*) and cue presentation occurring 6.5 sec before (marked as (**C**). For respiration, the area under the inspiration curve (AUC) is also highlighted, consistently with the analysis described in the methods section. Differential responses between stimuli with high and low unpleasantness are highlighted consistently with the analysis reported in the Results section: ***for differences at p < 0.001; **p < 0.01; *p < 0.05. “Expir.” refers to expiration, whereas “Inspir.” refers to inspiration.

**Figure 5:**
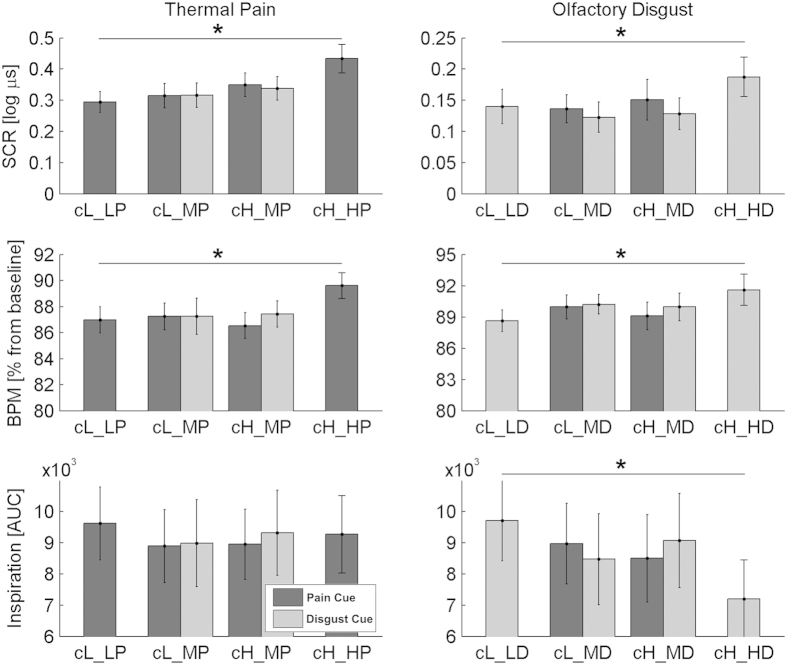
Average Skin Conductance Response (SCR), Heart Rate (HR), and inspiration volume (AUC) associated with the stimulation periods. Error bars represent standard errors of the mean. Axis and bar codes are similar to those in Fig. 2. *refer to conditions eliciting differential autonomic responses in the reference trials (full details in the text).

As a manipulation check, again, we first focused on the “reference” trials with correct predictive cues (*cHD_HD, cLD_LD, cHP_HP, cLP_LP*), in order to verify that the most unpleasant stimulations were associated with clear differential physiological responses. We therefore run, for each physiological parameter, a repeated-measure ANCOVA with Modality (Painful, Disgusting) and Unpleasantness (High, Low) as fixed factors, and the post-experimental evaluation of beliefs about the cue (see above) as a covariate. For both SCR and HR, this analysis revealed a main effect of Unpleasantness (Fs_(1,16)_ ≥ 16.98, Ps ≤ 0.001), indicating enhanced autonomic responses to stimuli of high (relative to low) unpleasantness. This general unpleasantness effect was also observed when focusing separately on each of the two modalities (*cHP_HP* *>* *cLP_LP*, ts_(17)_ ≥ 3.07, Ps ≤ 0.007; although for disgust this was true only without correcting for multiple comparisons: *cHD_HD* *>* *cLD_LD*, ts_(17)_ ≥ 2.35, Ps ≤ 0.031; see Figs 4 and 5, and Table 1). Furthermore, the SCR was associated with a main effect of Modality and a Modality*Unpleasantness interaction (Fs_(1,16)_ ≥ 8.45, Ps ≤ 0.010), essentially reflecting enhanced SCR for thermal (relative to olfactory) stimuli, especially when highly unpleasant (see Fig. 4 and Table 1). In a similar fashion, the analysis of inspiration volume revealed a main effect of Modality and an interaction of Modality*Unpleasantness (Fs_(1,16)_ ≥ 8.77, Ps ≤ 0.009), reflecting a decreased inspired air volume in olfactory (relative to thermal) stimuli, especially when highly unpleasant (see Figs 4 and 5, and Table 1). No other significant effects were found for any physiological measure (Fs_(1,16)_ ≤ 1.09, *n.s*). These data thus further validate our paradigm by demonstrating significant physiological reactions to high intensity pain and high intensity disgust on the “reference” trials.

We next analysed the critical medium trials of interest and run, separately for each physiological measure, an ANCOVA with Stimulus Modality (Painful, Disgusting), Cue Unpleasantness (High, Low) and Cue Modality (Pain, Disgust) as fixed factors, plus individual belief scores concerning the cue as covariate. In line with our results for the “reference” trials, the SCR data revealed a significant main effect of Stimulus Modality (F_(1,16)_ = 32.20, P = 0.00004), reflecting larger responses to thermal, as opposed to olfactory, events. Interestingly, the analysis of HR also revealed a main effect of Modality, but in this case olfactory stimuli were those associated with larger responses (F_(1,16)_ = 15.79, P = 0.001; see Table 1). More critically for our purpose, the analysis of SCR revealed a main effect of Cue Unpleasantness (F_(1,16)_ = 9.27, P = 0.008), reflecting significantly enhanced SCR when the identical medium- stimulation followed a high cue rather than a low cue (see Fig. 5 and Table 1). No other effects were found for SCR and HR measures, and no significant effect was associated with the inspiration volume (Fs_(1, 16)_ ≤ 5.77, n.s). Please note that all the effects associated with physiological measures described above could also be observed by removing the covariate from the analysis.

### Emotional response to the cue

**Figure 6:**
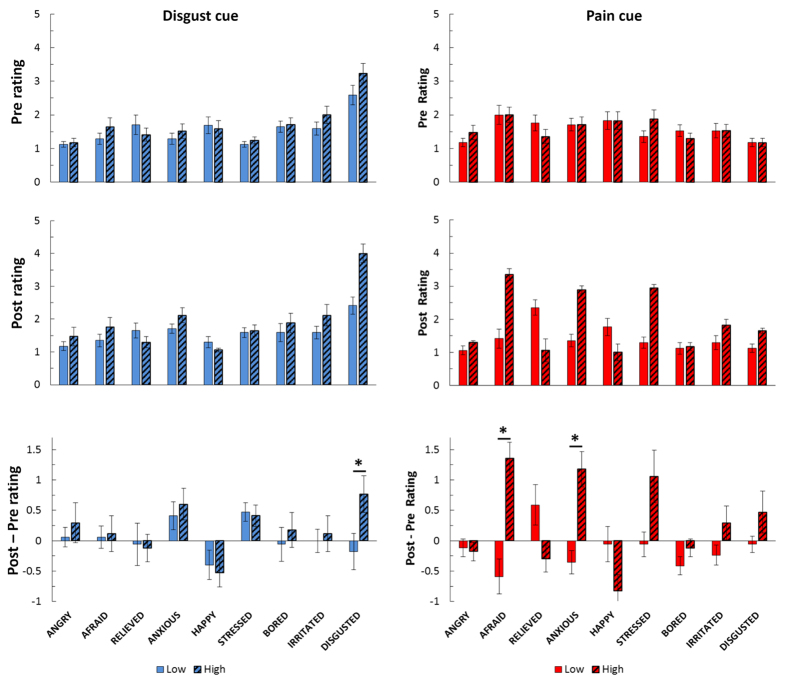
Emotional response to the different types of cue collected before (top row) and after the experimental session (middle row). Additionally, differential value (post-pre) are also displayed in the bottom row. For each row, rating values (averaged across 18 subjects) are plotted along 9 distinct emotional categories (anger, fear, relief, anxiety, happiness, stress, boredom, irritation, and disgust), on a scale from 1 (not at all) to 5 (absolutely). Ratings of pain (left column) and disgust (right column) cues are displayed in separate subplots and different colours. *refer to significant differences between cue of high vs. low unpleasantness in each modality. Error bars refer to standard errors of the mean.

Finally, we analysed emotional ratings for the cue itself. To assess the effect exerted by our experimental manipulation on the emotional judgment of each cue, we calculated the difference between the ipsatized scores^37^ after vs. before the experimental session, to remove any idiosyncratic/ pre-existing emotional effect independent from our task (see Fig. 6). These differential ratings were then compared between the high and low cues to test for any emotional modulation induced by expectations of events of high vs. low unpleasantness for each modality. For both pain and disgust, we considered reliable effects those associated with a p-value ≤ 0.0055 (corresponding to an error of 0.05, Bonferroni-corrected for the 9 emotion categories). With this threshold, we found that high pain cues (*cHP* > *cLP*) were associated with selective increase in fear (*t*_(16)_ = 4.94, P = 0.0001) and anxiety (*t*_(16)_ = 5.09, P = 0.0001), whereas high disgust cues (*cHD* > *cLD*) were associated with increased disgust (*t*_(16)_ = 4.17, P = 0.0007). No other emotional categories exceeded this threshold. These data therefore corroborate the fact that, despite the matched unpleasantness between pain and disgust, the expectation of different modalities evoked distinct emotional experiences in our participants, with high pain cues eliciting greater fear and anxiety, but high disgust cues eliciting greater disgust. Interestingly, visual inspection of Fig. 6 shows a consistent pre- vs. post increase in anxiety ratings also for the disgust cu; however this effect appeared in both the high and low disgust conditions, suggesting that it did not relate to the expectation of disgust specifically, but of olfactory events more generally.

## Discussion

Our study investigated whether expectation of pain or disgust elicits only specific sensory representations of the upcoming event or a representation of its affective unpleasantness, shared with other aversive experiences from different modalities. We designed a task where cues highly predictive of painful or disgusting events were followed by either a thermal or an olfactory stimulus. We found that cues predicting pain biased the subjective evaluation of subsequent thermal stimuli and induced more negative ratings despite identical temperature (in keeping with earlier findings^5^,^6^,^7^,^8^,^9^). Moreover, we found that cues predicting disgust produced similar biases in the subsequent evaluation of olfactory stimuli, extending these expectancy effects to the olfactory modality. Most importantly, we found that pain and disgust cues also affected the evaluation of stimuli from the opposite modality, demonstrating cross-modal expectation effects and suggesting a partially common representation. However, cross-modal influences were weaker than those obtained when the cue was consistent with the stimulation modality. Finally, expectancy effects also had an impact on autonomic arousal as measured by electrodermal activity, with larger SCR when the same stimulations were preceded by a high-unpleasantness (as opposed to low-) cue. Overall, our data indicate that representations recruited during the expectation of upcoming painful or disgusting events may contain both modality-specific and cross-modal information, with the latter presumably related to the affective (unpleasant) value of the stimulus coded in a modality-independent manner.

### Common and dissociated responses to pain and disgust

Pain and disgust are multi-component experiences with distinct properties, as they rely on different sensory input pathways (nociceptive vs. olfactory) and elicit divergent behaviours (e.g., fight/flight vs. regurgitation/vomit-like reactions^38^). However, despite their differences, there also entail a complex affective reaction that is partly shared across the two modalities. To pinpoint affective response to these two kinds of aversive experiences, we first compared trials in which high- and low- unpleasant stimulations were preceded by a consistent cue. These trials provided a reliable “reference” point for both the subjective evaluations and objective physiological responses, because our experimental procedure was carefully constrained to obtain olfactory and thermal stimuli with comparable levels of unpleasantness.

We found both unpleasant thermal and olfactory stimuli evoked (relative to their controls) not only more negative ratings (Fig. 2) but also larger SCRs and HR^36^,^39^,^40^ (Fig. 5 and Table 1). Because all stimuli were delivered in a cued sniff paradigm (see methods), HR during stimulation was modulated by respiration (see Fig. 4: 0–5 s post stimulus onset), but critically it also increased when the stimuli were unpleasant (between 5–8 s; see also^36^), regardless of their modality. Moreover, such unpleasantness effect of HR is classically linked to defence reactions that permit the avoidance of unpleasant stimulation (e.g., when rejecting noxious matter^41^,^42^). Thus, our physiological data neatly converge with our subjective rating data to indicate common patterns of aversive response to both unpleasant odours and painful temperatures.

Interestingly, however, autonomic responses also showed dissociations between modalities, with SCRs more strongly modulated by thermal than olfactory stimuli (regardless of their unpleasantness), but an opposite pattern for inspiration volume. In particular, the Modality*Unpleasantness interaction observed for SCR suggests larger arousal evoked by pain than disgust (although disgust also increased galvanic responses, but to a lesser extent; see Fig. 5 and Table 1). Likewise, the same interaction observed for inspiration volumes is explainable as an olfactory-specific decrease for stimuli of high (relative to low) unpleasantness. Although to our knowledge no study has directly compared autonomic responses for equally unpleasant (according to explicit ratings) thermal and olfactory stimuli, we can provide two (non mutually exclusive) explanations for the differences observed between modalities. One hypothesis might refer to idiosyncratic properties of each physiological measure leading to greater sensitivity for one or the other modality: e.g., galvanic responses might be more sensitive to the affective load of thermal stimuli. This possibility accords with previous evidence showing that events from different sensory channels trigger distinct physiological responses, including pain^43^ and disgust^44^. Alternatively, SCRs might be more closely related to arousal or emotion dimensions associated with pain (but not disgust), independent from the effects of unpleasantness, as suggested by a recent meta-analysis which proposed that the autonomic system may generate a set of multiple responses that differ in across the basic emotions^45^. In line with this interpretation, the emotional ratings gathered from the pre- and post-test debrief questionnaire, which required participants to report emotions experienced when viewing the different types of cues. When comparing these emotion judgments before and after the experimental session, we found that high pain cues were associated with higher fear and anxiety, whereas high odor cues evoked stronger disgust but not fear. Taken together, the analyses of both the ratings and physiological measures support a multi-componential emotional response to painful and disgusting events, with both shared and independent aspects.

### Expectancy of Pain and Disgust

The main focus of our study was to examine trials with thermal and olfactory stimuli at a fixed (medium) unpleasantness level but when preceded by different kinds of cue. This allowed us to identify effects that were purely driven by expectancy, whilst minimizing any bottom-up confound. Our results accord with previous findings that the subjective pain experience evoked by identical thermal stimulus is strongly influenced by expectations^5^,^6^,^7^,^8^,^9^. Furthermore, here we found similar biases for olfactory stimuli. Such expectancy effect on olfaction add to studies showing that the subjective evaluation of odours may be biased by experimental manipulations of odour labels^46^,^47^,^48^, personal beliefs^49^,^50^, or social cues^50^,^51^. Our study extends these data, not only by showing that comparable expectancy effects arise for both thermal and olfactory stimuli, but also by demonstrating for the first time cross-modal expectancy effects between pain and disgust, in addition to modality-specific modulations. Thus, trials with medium intensity stimuli revealed that the subjective affective evaluation of both thermal and olfactory events was biased by a cue predicting their unpleasantness, even from an opposite modality.

Crossmodal effects associated with pain, olfaction, or both have already been observed in other situations. For instance, olfaction can be modulated by gustation (possibly through integrated representations of flavour)^52^,^53^ or by vision^54^,^55^. A similar effect was documented between pain perception and vision^56^,^57^. Furthermore, pain unpleasantness can be affected by the valence of a simultaneously presented odour^15^,^16^. Our data converge with these studies and suggest that pain and disgust might be processed at least partly through a representation that is not specific to a given modality, but encodes aversive meaning from different sensory channels, linked to different behavioural responses^14^,^38^,^58^. Most importantly, these results also extend previous work by showing that pain and disgust are processed in a crossmodal fashion, not only during the stimulus delivery, but also during stimulus expectation. Thus, the expectancy of pain and disgust might elicit information about the negative/aversive affective value of the upcoming event, regardless of the consistency in sensory modality between the cue and the stimulus. These subjective effects were paralleled by our SCR results which showed a general enhancement on medium trials whenever a cue of high (relative to low) unpleasantness was presented.

Moreover, brain imaging studies indicate that neural response elicited by the experience of pain^11^,^12^ and disgust^18^,^23^,^24^ partially overlap in many brain regions (amygdala, anterior insula and middle cingulate cortex, etc.). These regions constitute key components of a distributed brain network (the *salience network*) involved in the detection of homeostatically relevant inputs and promotion of appropriate behavioural adjustment^20^,^21^. The cross-modal expectancy effects observed in our study could reflect an engagement of the salience network equally responsive to cues predicting pain or disgust. Such shared expectancy effects would provide further support to the hypothesis that these brain areas may encode predictive feeling states that guide behavioural choices and adaptation^59^. Future neuroimaging studies should shed more light on the neural substrates of these effects.

Interestingly, however, although cross-modal expectancy effects were robust and reliable, their magnitude was significantly smaller than those associated with consistent trials (i.e. when cues predicted the correct stimulation modality). This within-modality enhancement suggests that the cues also elicited a representation of sensory-specific properties of the upcoming event. Moreover, this enhancement was larger in participants who reported stronger conscious reliance on the cue during anticipation prior tostimulation. A plausible interpretation is that, besides the supramodal signals of negative predictive states subserved by shared brain networks, the processing of pain and disgust cues also modulated activity within sensory regions more selectively engaged by either noxious (e.g., posterior insula, somatosensory cortex) or olfactory stimuli (orbitofrontal and entorhinal cortex). Such anticipatory increase could then amplify the responsiveness of these sensory areas to the subsequent stimulation. Future neuroimaging studies will be needed to verify this interpretation, by measuring both the distinct and common patterns of neural activity associated with the anticipation of pain and disgust.

### Limitations of the study

Like in previous studies using similar pain-expectancy paradigms^6^,^8^,^9^, our visual cues did not elicit changes in autonomic activity during the expectancy period (see Supplementary Materials), although they affected the response evoked by the subsequent thermal or olfactory stimuli. This may seem at odds with other studies that show how stimuli predicting pain (e.g., videos of approaching needles^60^) elicit sustained responses. It is possible that the autonomic changes associated with the expectancy period might partially depend on arousing properties of the stimulus used as cue, or the distinctiveness of the alternative outcomes, rather than on the kind of information conveyed by the cue.

Furthermore, one common limitation of studies employing olfactory stimuli lays in the fact that people often modify their breathing during the occurrence of unpleasant odors, which can potentially interfere with perceptual and emotional judgments of odors. We used a cued-sniffing paradigm^36^ in order to minimize these biases by forcing participants to inspire a comparable amount of air in all trials. Analysis of respiration data confirmed that this was indeed the case on the critical medium trials, regardless of the cue and of the subsequent stimulation. However, this was not the case for the highly-unpleasant reference trials which corresponded to the most unpleasant odors and were associated with smaller inspiration amplitudes compared to low-unpleasant and positive odors (see Figs 4 and 5 and Table 1). It is therefore possible differential respiration patterns could have affected some of the effects observed for these trials. However, these reference trials only served as a “baseline” condition to establish the effect of aversive stimuli, and no such respiration difference occurred for the critical medium-intensity stimulations where expectancy biases were examined.

### Conclusions

In conclusion, our study provides novel and compelling evidence that expectations of high pain or high disgust events induce negative biases that make a subsequent stimulus feel more unpleasant, regardless of whether this stimulus appears in the same or another modality. These findings support the notion of a shared representation for aversive prediction signals across modalities, consistent with overlapping brain networks engaged by aversive or salient stimuli of different sensory modalities.

## Additional Information

**How to cite this article**: Sharvit, G. *et al.* Cross-modal and modality-specific expectancy effects between pain and disgust. *Sci. Rep.* **5**, 17487; doi: 10.1038/srep17487 (2015).

## Supplementary Material

Supplementary Information

## Figures and Tables

**Table 1:** Average Ratings, SCRs, HR and inspiration volume associated with each experimental conditions (bracket values refer to standard error of the mean).

Trial type	Ratings [mean]	SCR [log μS]	BPM [% from baseline]	Inspiration [AUC]
*Reference trials*				
cLP_LP	−7.88 (1.36)	0.29 (0.03)	86.9 (1.0)	9941.7 (1176.5)
cLD_LD	−5.94 (1.17)	0.14 (0.03)	88.6 (1.0)	9828 (1292.4)
cHP_HP	−38.10 (1.66)	0.43 (0.04)	89.6 (0.9)	9642.9 (1242.7)
cHD_HD	−35.78 (2.62)	0.19 (0.03)	91.6 (1.5)	7522 (1297.4)
*Medium-consistent trials*				
cLP_MP	−15.76 (2.25)	0.31 (0.04)	87.2 (1.0)	8898.4 (1131.8)
cLD_MD	−13.91 (1.90)	0.12 (0.02)	90.2 (0.9)	8673 (1433.6)
cHP_MP	−25.77 (2.81)	0.35 (0.04)	86.5 (0.9)	9519 (1174.2)
cHD_MD	−23.74 (2.58)	0.13 (0.02)	89.9 (1.3)	9000.7 (1407)
*Medium-inconsistent trials*				
cLD_MP	−15.39 (2.07)	0.31 (0.04)	87.2 (1.3)	9200.2 (1285.4)
cLP_MD	−12.38 (1.80)	0.14 (0.02)	89.9 (1.1)	9130.4 (1252.4)
cHD_MP	−24.08 (2.77)	0.38 (0.04)	87.4 (1.0)	9473.5 (1352.5)
cHP_MD	−20.55 (2.45)	0.15 (0.03)	89.0 (1.3)	8532.1 (1430.2)
