# Erratum: Cross-modal and modality-specific expectancy effects between pain and disgust

**DOI:** 10.1038/srep21219

**Published:** 2016-02-17

**Authors:** Gil Sharvit, Patrik Vuilleumier, Sylvain Delplanque, Corrado Corradi-Dell’Acqua

Scientific Reports 5: Article number: 17487; 10.1038/srep17487 published online: 12032015; updated: 02172016.

The original version of this Article contained a typographical error in the spelling of the author Corrado Corradi-Dell’Acqua which was incorrectly given as Corrado Corradi-Dell’ Acqua. This error has been corrected in both the PDF and HTML versions of the paper.

