# Supplementary material for: Cross-modal and modality-specific expectancy effects between pain and disgust
Source: Sci Rep. 2015 Dec 3;5:17487. doi: 10.1038/srep17487 (PMC4668356; doi:10.1038/srep17487)
Supplement: Supplementary Information [file srep17487-s1.pdf]

Supplementary Materials for the article:

"Cross-modal and modality-specific expectancy effects between pain and disgust"

by Gil Sharvit, Patrik Vuilleumier, Sylvain Delplanque & Corrado Corradi-Dell'Acqua

Preselection of odorants

In this preselection task, all 12 odours (plus 13th odourless control) were delivered as follows. Each trial began with a 1 second (s) fixation cross at the centre of the computer screen; then the instruction "Breath-out" was presented together with a numerical 3 s countdown. During the countdown, participants were instructed to expire and empty their lungs. When the countdown reached 0, the instruction "Breathe-in" was presented concurrently with the delivery of the odorant (2 s). This trial structure[1] allowed to minimize the intra- and inter participant breathing pattern variability and to synchronize the respiration cycle with the odorant delivery regardless of its nature. After each stimulus, a visual analogic scale was presented. Participants were asked to rate the degree of subjective unpleasantness/pleasantness evoked by the odorant by marking the corresponding position on the scale with a mouse device held in their right hand. The 13 stimuli were presented twice in an equally distributed and pseudorandomized order. This odorant selection session lasted approximately 15 minutes.

Preselection of thermal stimuli

In line with previous studies on pain, individual temperatures were determined through a double random staircase (DRS) algorithm[2,3]. However, unlike these studies focusing on thresholds that elicit self-reported pain, our approach searched for temperatures associated with unpleasantness ratings (measured with the same visual analog scale as for the odorants selection session) that were comparable to those reported for the highly unpleasant odours. Our DRS procedure selected a given temperature on each experimental trial according to the previous response of the participant. Trials rated as more unpleasant than the given cut-off (selected in a subject-specific way, from ratings for the highly unpleasant odour) led to a subsequent lowered temperature in the next trial; whereas trials rated as less unpleasant than the given cut-off led to a subsequent higher temperature. This resulted in a sequence of temperatures that rapidly ascended towards, and subsequently converged around, a subjective unpleasantness threshold, which was in turn calculated as the average value of the first 4 temperatures leading to a direction change in the sequence. In order to avoid participants anticipating a systematic relationship between their rating and the subsequent temperature, two independent staircases were presented randomly. Initial thermal stimulations for the two staircases were 41°C and 43 °C. Within each staircase, stimulus temperatures increased or decreased with steps of 3°C, while smaller changes (1°C) occurred following direction flips in the sequence. None of our subjects was stimulated at temperature larger than 50°C.

The thermal stimuli were delivered in the following way: participants first saw a 1 s long fixation-cross, followed by the text string "Temperature is changing" and concomitant delivery of the heat stimulation. Each thermal event was composed of 3 s of rise time, 2 s of plateau at the target-temperature, and 3 s of return to baseline (37°C). The speed of the temperature rise and the temperature return was automatically adjusted according to the plateau in order to maintain both a rise time and a return time of approximately 3 s each. The unpleasantness scale was presented just after the 2 s of plateau stimulation, when the temperature started to return to baseline, and lasted until participant provided a response.

The present DRS approach was employed to determine temperatures eliciting three distinct levels of unpleasantness (corresponding to different levels of pain): low, moderate, and high. This approach led to a highly unpleasant temperature, which varied on a participant-by-participant basis, but converged around the average value of 46.85°C (SEM 0.5). On the basis of this temperature, we selected two additional temperatures associated with medium (1/1.5°C less than the highly unpleasant temperature, corresponding to an average of 45.62°C, SEM 0.51) and low unpleasantness (2/3°C less than the highly unpleasant temperature, corresponding to an average 44.39°C, SEM 0.53). This session lasted approximately 10 minutes, and was repeated twice (one for each of participant's leg).

Analysis of physiological responses during the expectations period

As for the case of the stimulus events we analysed the SCR and average HR associated with the expectancy period between the presentation of the cue and the stimulus delivery. In particular, we measured the artefact-free amplitude of SCRs evoked by cues, by considering a reliable SCR amplitude the one exceeding a threshold of 0.02 µS that started between 1 and 4 s after the cue presentation, and peaked in the period between this onset time and the delivery of the stimulation. The resulting amplitude value was log-transformed. As for the HR, differentially from the analysis of stimulus induced physiological changes in which the respiration cycle was constrained, we calculated average HR from the cue presentation to the delivery of the stimulus.

For each of these measures, we run a repeated measures ANCOVAs with Cue Unpleasantness (High, Low) and Cue Modality (Pain, Disgust) as fixed factors and individual reliance of the cue as covariate. These analyses led to no significant effects ($Fs_{(1,16)} \leq 1.51$, n.s.).

References

1. Delplanque, S. *et al.* Sequential unfolding of novelty and pleasantness appraisals of odors: evidence from facial electromyography and autonomic reactions. *Emotion* **9,** 316–28 (2009).

2. Eisenberger, N. I. *et al.* Attachment figures activate a safety signal-related neural region and reduce pain experience. *Proc. Natl. Acad. Sci. U. S. A.* **108,** 11721–6 (2011).

3. Gracely, R. H., Lota, L., Walter, D. J. & Dubner, R. A multiple random staircase method of psychophysical pain assessment. *Pain* **32,** 55–63 (1988).
